# Co-detection *of Bordetella pertussis* and other respiratory organisms in children hospitalised with lower respiratory tract infection

**DOI:** 10.1038/s41598-020-73462-w

**Published:** 2020-10-02

**Authors:** Rudzani Muloiwa, Felix S. Dube, Mark P. Nicol, Gregory D. Hussey, Heather J. Zar

**Affiliations:** 1 Department of Paediatrics and Child Health, Groote Schuur Hospital, University of Cape Town, Anzio Road, Observatory, Cape Town, South Africa; 2 Department of Molecular and Cell Biology, Faculty of Science, University of Cape Town, Cape Town, South Africa; 3 Institute of Infectious Disease and Molecular Medicine, University of Cape Town, Cape Town, South Africa; 4 Division of Infection and Immunity, School of Biomedical Sciences, University of Western Australia, Crawley, Australia; 5 Division of Medical Microbiology, Faculty of Health Sciences, University of Cape Town, Cape Town, South Africa; 6 Vaccines for Africa Initiative, Division of Medical Microbiology, University of Cape Town, Cape Town, South Africa; 7 SA-MRC Unit On Child and Adolescent Lung Health, University of Cape Town, Cape Town, South Africa; 8 Department of Paediatrics and Child Health, Red Cross War Memorial Children’s Hospital, University of Cape Town, Cape Town, South Africa

**Keywords:** Bacterial infection, Respiratory tract diseases

## Abstract

Multiple potential pathogens are frequently co-detected among children with lower respiratory tract infection (LRTI). Evidence indicates that *Bordetella pertussis* has an important role in the aetiology of LRTI. We aimed to study the association between *B. pertussis* and other respiratory pathogens in children hospitalised with severe LRTI, and to assess clinical relevance of co-detection. Nasopharyngeal (NP) swabs and induced sputa (IS) were tested with a *B. pertussis* specific PCR; additionally, IS was tested for other pathogens using a multiplex PCR. We included 454 children, median age 8 months (IQR 4–18), 31 (7%) of whom tested positive for *B. pertussis*. Children with *B. pertussis* had more bacterial pathogens detected (3 versus 2; P < 0.001). While *B. pertussis* showed no association with most pathogens*,* it was independently associated with *Chlamydia pneumoniae, Mycoplasma pneumoniae* and parainfluenza viruses with adjusted risk ratios of 4.01 (1.03–15.64), 4.17 (1.42–12.27) and 2.13 (1.03–4.55), respectively. There was a consistent increased risk of severe disease with *B. pertussis.* Patterns indicated even higher risks when *B. pertussis* was co-detected with any of the three organisms although not statistically significant. Improving vaccine coverage against *B. pertussis* would impact not only the incidence of pertussis but also that of severe LRTI generally.

## Introduction

Lower respiratory tract infection (LRTI) is responsible for a large burden of morbidity and mortality in children each year^1^. Current understanding is that the aetiology of LRTI may frequently be polymicrobial, with various combinations of viral and bacterial pathogens implicated in the pathogenesis^2–5^. *Bordetella pertussis*, the organism that causes whooping cough, is one of the organisms strongly associated with LRTI in children, however the role of other organisms in the pathogenesis of LRTI is not well-understood^4^.

Effective vaccines against *B. pertussis* have been available since the 1940s. Initially these were of the whole cell type (wP) but have since the 1990s been superseded by acellular vaccines (aP) mainly in high income countries^6^. The introduction and wide use of vaccines have markedly reduced the burden of pertussis over the last six decades, but there is strong evidence that pertussis has resurged all over the world in recent years, bringing into focus *B. pertussis* as an important respiratory pathogen in the aetiology of respiratory illness, including LRTI^7,8^.

Although the presence of *B. pertussis* has been described together with other organisms that are potential co-pathogens in individuals with LRTI, most studies have focused on the role of viruses in respiratory tract co-infections^9^. As a result, potential interactions between *B. pertussis* and other organisms remain poorly understood^10–12^. The organism, *B. pertussis,* produces several toxins that aid the organism in evading the immune system, assisting it to establish infection on the respiratory epithelium^13,14^.

We hypothesise that the conditions produced by *B. pertussis* toxins not only create a conducive environment for *B. pertussis* itself but may facilitate the colonisation or infection of the respiratory tract epithelium by other bacteria or viruses. In this study, we aimed to investigate whether the detection of *B. pertussis* in children hospitalised for LRTI was associated with co-detection of other potential respiratory pathogens. We also explore whether there was an association between co-detection and clinical severity and outcome.

## Methods

### Recruitment and specimen collection

Methods for sampling as well as inclusion criteria have been described elsewhere^15^. Briefly, the study recruited inpatient children seen at a referral hospital, Red Cross War Memorial Children’s Hospital (RCH) in Cape Town, South Africa over a one-year period (September 2012 to September 2013). Children were recruited if they presented with cough and WHO defined age specific tachypnoea, or apnoea, and were ill enough to warrant admission. Only children whose legal guardians were present to give written consent were enrolled. As the study intended to describe co-infection involving community acquired organisms, we excluded children who had been in touch with the health care services in the preceding two weeks in order to minimise health care-associated infection.

To assess the severity of respiratory symptoms, the presence of chest indrawing was noted. In addition, all children had pulse oximetry to assess for oxygen saturation. A cut-off of < 94% was used to define hypoxaemia in children at sea level^16–18^.

A detailed history of the current illness was collected, and participants underwent testing for HIV infection. The diagnosis of HIV infection was made for children less than 18 months of age if they tested positive for two HIV polymerase chain reaction (PCR) tests (COBAS AmpliPrep/COBAS Taqman HIV-1, Roche Molecular Diagnostics, Pleasanton, CA, USA). For children above 18 months of age, HIV infection was diagnosed on the basis of two positive ELISA tests using two different assays (Architect HIV Ag/Ab Combo, Abbott Diagnostics, Wiesbaden; and Enzygnost Anti-HIV 1/2 Plus, Siemens/Dade Behring, Erlange, sequentially).

All children had anthropometry (weight and height), performed at enrolment by trained study staff. Nutritional status was classified using WHO weight for age z-scores (WAZ). Children were classified as moderate to severely malnourished if their weight for age fell below − 2 z-scores.

The children’s vaccination status was sourced from their handheld clinic booklets. The primary schedule according to the South African Expanded Program on Immunisation, in addition to other vaccines, contains an acellular (aP) vaccine against *B. pertussis* combined with that against *Haemophilus influenzae type b* at 6, 10 and 14 weeks (with a booster at 18 months), and vaccination with 13-valent pneumococcal conjugate (PCV13) vaccine at 6 and 14 weeks of age (with a booster at 9 months)^19^.

A nasopharyngeal (NP) swab was collected first after which an induced sputum (IS) specimen was collected on enrolment as previously described^20^. Molecular diagnostic testing was carried out on batched specimens as described below. No blood culture was done as part of the study.

### Laboratory methods

#### Diagnosis of *Bordetella pertussis*

To diagnose *Bordetella pertussis* infection*,* PCR specific for IS*481* for *Bordetella* species was conducted on both NP and IS specimens with a validated commercial kit (Roche LightMix, Basel) using previously published primers^21^. *Bordetella holmesii* (defined as IS*481*+ and hIS*1001*+) which shares the same IS*481* target as *B pertussis* was excluded by further testing all IS*481* positive specimens for the presence of insertion site hIS*1001*^22^.

#### Diagnosis of co-infections

The FTDResp 33 multiplex real-time PCR assay (Fast-Track Diagnostics, Esch-sur-Alzet, Luxembourg) was used to identify presence of a range of viruses and bacteria as well as *Pneumocystis jirovecii* on IS. As the study was designed specifically to study the epidemiology of pertussis in this population, for the analysis, *B. pertussis* results from LightMix for *B. pertussis* (rather than those from FTDResp 33) were used as our assessment indicated that the assay had better sensitivity for *B. pertussis* than Fast-Track, although both employ the same targets.

### Statistical analysis

We used percentages to depict proportions of study participants with organisms detected from respiratory specimens. Continuous data were tested for normality and summarized as medians with interquartile ranges (IQR) or means and standard deviations (SD) as appropriate. The difference in total numbers of organisms detected in participants with and without confirmed *B. pertussis* was compared using Student’s t-test. χ^2^ or Fisher’s exact tests were used to assess the strength of association between infection with *B. pertussis* and each co-pathogen. All associations at a two-tailed P < 0.1 were further analysed adjusted for sex, age and HIV status as potential confounders. Generalised linear modelling using Poisson regression with robust error variance was used to estimate adjusted relative risks (aRR) and their 95% confidence intervals in a multivariable analysis. Severity of clinical disease and outcomes were further analysed stratified by a combination of pertussis status and organisms showing strong association with pertussis. Continuous data were tested for normality and comparisons between groups were made with the appropriate test for parametric or non-parametric data as indicated. Statistical significance was set at a two-sided P < 0.05. All analyses were carried out using *Stata Statistical Software Release 16* (StataCorp LP, College Station, TX).

### Statement on ethics approval

Prior approval for the study was obtained from the Human Research Ethics Committee of the Faculty of Health Sciences of the University of Cape Town; reference: 371/2011. Written informed consent was sought and received from the parent or legal guardian of each child in order for the child to participate in the study. All methods were carried out in accordance with the relevant guidelines and regulations.

## Results

### Baseline data

Four hundred and sixty children were enrolled. Six children, including four whose IS could not be collected and two whose IS were lost prior to processing during transportation or storage, were excluded, providing 454 participants with sufficient data for analysis, of which 253 (55.7%) were male.

The median age of the children was 8 (IQR 4 -18) months. HIV infection was confirmed in 19 (4.2%) of the children. History of asthma or eczema was present in two children, while 26 (5.9%) of the caregivers (all mothers to the children) reported a history of asthma. Nine children (2.0%) did not have their immunization records with them. Of the 445 (98.0%) with known vaccination status, 321(72.1%) were up to date with pertussis and *Haemophilus influenzae type b* vaccine doses for age, while 418 (93.9%) had received at least one dose of the combination. Similarly, 312 (70.1%) were up to date with PCV13 doses for age with 385 (86.5%) having received at least one dose of the vaccine. Baseline characteristics of the study group are summarized in Table 1.

**Table 1:** Baseline characteristics of study participants.

Baseline character	*N* = *454*
**Age in months [**Median (interquartile range)]	8 (4–18)
	**n (%)**
**Male sex**	253 (55.7.)
**Pertussis/H. influenza type b vaccines doses**	
0	31 (6.8)
1	59 (13.0)
2	61 (13.4)
≥ 3	294 (64.8)
Unknown	9 (2.0)
**Pneumococcal conjugate vaccine (PCV13) doses**	
0	60 (13.2)
1	108 (23.8)
2	139 (30.6)
3	138 (30.4)
Unknown	9 (2.0)
**HIV infected**	19 (4.2)
**Nutritional status**	
Normal	384 (90.8)
WAZ ≤ -2	39 (9.2)
**History of prematurity**	77 (17.0)
(Gestational age < 37 weeks)	
**Presence of a home cigarette smoker**	62 (13.7)
**Breast-feeding history**	
Never breastfed	58 (12.8)
Breastfed < 4 months	320 (70.5)
Breastfed > 4 months	76 (16.7)
**Pre-hospital antibiotic**	
Yes	153 (36.2)
No	270 (63.8)
**Oxygen saturation < 94% in room air**	70 (15.4)
**Chest indrawing**	380 (83.7)
**Confirmed Bordetella pertussis**	31(6.8)

WAZ = World Health Organisation weight for age Z-score < –2.

### Confirmed *Bordetella pertussis* infection

PCR for insertion site IS*481* was positive in 16 NP specimens and 25 IS specimens on LightMix. Ten participants had a positive PCR on both NP and IS specimens, therefore 31 (6.8%; 95% CI 4.7–9.6%) participants were confirmed as having *B. pertussis* infection. The *B. holmesii* insertion site hIS*1001,* was not identified in any of the IS*481* positive specimens. Only 10 (2.2%) samples, all also found to be positive on LightMix, were positive for *B. pertussis* on Fast-Track testing of IS samples.

The median age of children with confirmed *B. pertussis* was 8 (IQR 2–22) months while those testing negative had a median age of 8 (IQR 4–18) months; P = 0.559.

*B. pertussis* was isolated in 5 (18.5%) of the 27 who did not get any pertussis vaccine dose compared to 26 (6.2%) of the 418 who received at least one vaccine dose; P = 0.032.

### General description of PCR detected pathogens

In most participants (n = 412; 90.7%) both a viral and bacterial organism were co-detected from the IS specimen. Four hundred and forty-one (97.1%) participants had at least one virus identified from an IS specimen with 29 (6.4%) having only a virus detected. In 421 (92.7%) at least one bacterial species was identified, with 9 (2.0%) only having a bacterial species detected. Four (0.9%) of the 454 children did not have any organism (including *B. pertussis*) identified from their IS specimen.

**Figure 1:**
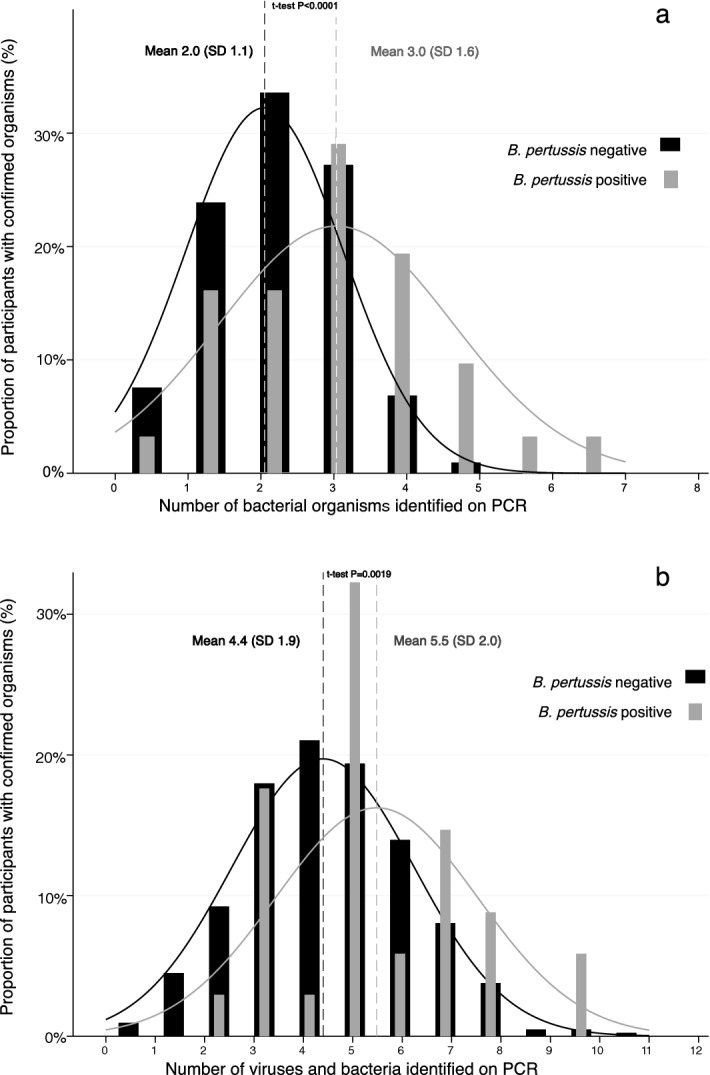
Distribution of number of bacteria (**a**) and bacteria + viruses (**b**) identified on polymerase chain reaction (PCR) in participants with and without Bordetella pertussis.

For all participants with confirmed *B. pertussis* infection, a minimum of two organisms were identified. The average number of viruses detected in children with and without confirmed pertussis were 2.5 (SD 1.4) and 2.3 (SD 1.3) respectively; P = 0.665. Children with confirmed pertussis had on average 3.0 (SD 1.6) different bacterial species detected while those without had 2.0 (SD 1.1); P < 0.001. When both bacterial and viral organisms were considered, the average number identified in pertussis positive participants was 5.5 (SD 2.0) and 4.4 (SD 1.9) in the pertussis negative group; P = 0.009 (Fig. 1).

The prevalence of specific organisms identified on IS in participants with and without confirmed pertussis is shown in descending order of frequency in Table 2.

**Table 2:** Association between *Bordetella pertussis* and other organisms isolated on IS (N = 454).

Pathogen	Total n (%)	*Bordetella pertussis* PCR n (%)		P value^#^
		Positive n = 31	Negative n = 423	
**Viral organisms**				
Cytomegalovirus	253 (55.7)	17 (54.8)	236 (55.8)	0.918
Rhinovirus	222 (48.9)	17 (54.8)	205 (48.5)	0.493
Respiratory Syncytial Virus	135 (30.7)	5 (16.1)	130 (30.7)	**0.086**
Adenovirus	117 (25.8)	9 (29.0)	108 (25.5)	0.667
Bocavirus	80 (17.6)	8 (25.8)	72 (17.0)	0.215
Enterovirus-parechovirus	80 (17.6)	7 (22.6)	73 (17.3)	0.453
Parainfluenza (1,2,3 4)	75 (16.5)	9 (29.0)	66 (15.6)	**0.052**
Metapneumovirus A & B	45 (9.9)	1 (3.2)	44 (10.4)	0.345
Coronavirus (43,63,229,hku)	35 (7.7)	3 (9.7)	32 (7.6)	0.723
Influenza (A, B, C)	28 (6.2)	0 (0.0)	28 (6.6)	0.244
**Bacterial organisms**				
*Moraxella catarrhalis*	295 (65.0)	18 (58.1)	277 (65.5)	0.403
*Streptococcus pneumoniae*	240 (52.9)	17 (54.8)	223 (52.7)	0.819
*Haemophilus influenzae*	231 (50.9)	18 (58.1)	213 (50.4)	0.407
*Staphylococcus aureus*	136 (30.0)	9 (29.0)	127 (30.0)	0.907
*Haemophilus influenzae B*	16 (3.5)	2 (6.5)	14 (3.3)	0.299
*Mycoplasma pneumoniae*	10 (2.2)	3 (9.7)	7 (1.7)	**0.025**
*Chlamydia pneumoniae*	7 (1.2)	2 (6.5)	5 (1.2)	**0.076**
**Fungal organism**				
*Pneumocystis jirovecii*	98(21.6)	8 (25.8)	90 (21.3)	0.554

IS = induced sputum. # Two-sided Fisher’s exact or Chi Square tests P-values; Bold Typeface = P < 0.1.

^¶^Organisms shown in descending order of total frequency for each pathogen group.

### Co-detection of *Bordetella pertussis* with specific organisms

Overall, *Moraxella catarrhalis* was the commonest bacterium identified with 295 (65.0%) samples testing PCR positive for the organism. *Mycoplasma pneumoniae* (P = 0.025) showed strong association with *B. pertussis* infection while *Chlamydia pneumoniae* (P = 0.076) displayed weaker evidence of association with *B. pertussis*. No other bacteria were significantly associated with confirmed pertussis (Table 2). Cytomegalovirus was the commonest virus, identified in 253 (55.7%) of the participants. Parainfluenza viruses (1, 2, 3 & 4) were weakly associated (P = 0.052) with the presence of *B. pertussis*, while Respiratory Syncytial Virus (RSV) was weakly associated with the absence of *B. pertussis* (P = 0.086). The other viruses did not show any association with *B. pertussis* infection (Table 2). *Pneumocystis jirovecii* was detected in 8 (25.8%) and 98 (21.6%) of participants with and without confirmed pertussis, respectively, P = 0.554.

After adjusting for potential confounding, two bacterial pathogens, namely *C. pneumoniae* (aRR 4.01 (95% CI 1.03–15.64) and *M. pneumoniae* (aRR 4.17 (95% CI 1.42–12.27) remained independently associated with confirmed *B. pertussis* infection*.* Parainfluenza viruses (aRR 2.13 (95% CI 1.03–4.55) was the one group of viruses that showed significant independent association with confirmed pertussis. Lack of strong association between the absence of *B. pertussis* and RSV remained unchanged after adjusting for potential confounding (Table 3).

**Table 3:** Risk of lower respiratory co-infection in children with confirmed *Bordetella pertussis* infection.

Co-infection	*Bordetella pertussis* PCR n (%)		RR (95% Confidence interval)	
	Positive n = 31	Negative n = 423	Crude	Adjusted^#^
*Chlamydia pneumoniae*	2 (6.5)	5 (1.2)	**4.40 (1.29–14.98)**	**4.01 (1.03–15.64)**
*Mycoplasma pneumoniae*	3 (9.7)	7 (1.7)	**4.75 (1.73–13.11)**	**4.17 (1.42–12.27)**
Parainfluenza (1,2,3,4)	2 (6.5)	4 (1.0)	2.07 (0.99–4.31)	**2.13 (1.03–4.55)**
*Respiratory syncytial virus*	5 (16.1)	130 (30.7)	0.45 (0.18–1.16)	0.45 (0.17–1.20)

RR = Relative risk; ^#^ Multivariable model adjusted for age in months, sex and HIV status; confidence intervals not overlapping the null value of 1 are shown in bold typeface.

### Clinical presentation and outcome

Only three (9.7%) of the 31 *B. pertussis* PCR positive cases were diagnosed clinically with pertussis. All 454 participants were discharged from hospital with no in-hospital deaths occurring in both the *B. pertussis* PCR positive and negative groups. Twelve (2.4%) children required a High Dependency Unit or Paediatric Intensive Care Unit admission; slightly higher frequency in children with confirmed *B. pertussis* with two out of 31 (6.5%) and 10 out of 423 (2.4%) in the positive and negative groups respectively; P = 0.194. Due to the small numbers, no further analysis was possible.

Hypoxaemia as indicated by oxygen saturation was noted in 70 (15.4%) children while chest indrawing was noted in 380 (83.7%). In general, children with confirmed pertussis showed higher frequencies of chest indrawing with 28 (90.3%) out of 31 compared to 352 (83.2%) of the 423 (P = 0.449) without pertussis. Similarly, there were 9 (29.0%) out of 31 compared to 61(14.4%) out of 423 (P = 0.039) showing hypoxaemia in children with and without *B. pertussis*, respectively.

**Figure 2:**
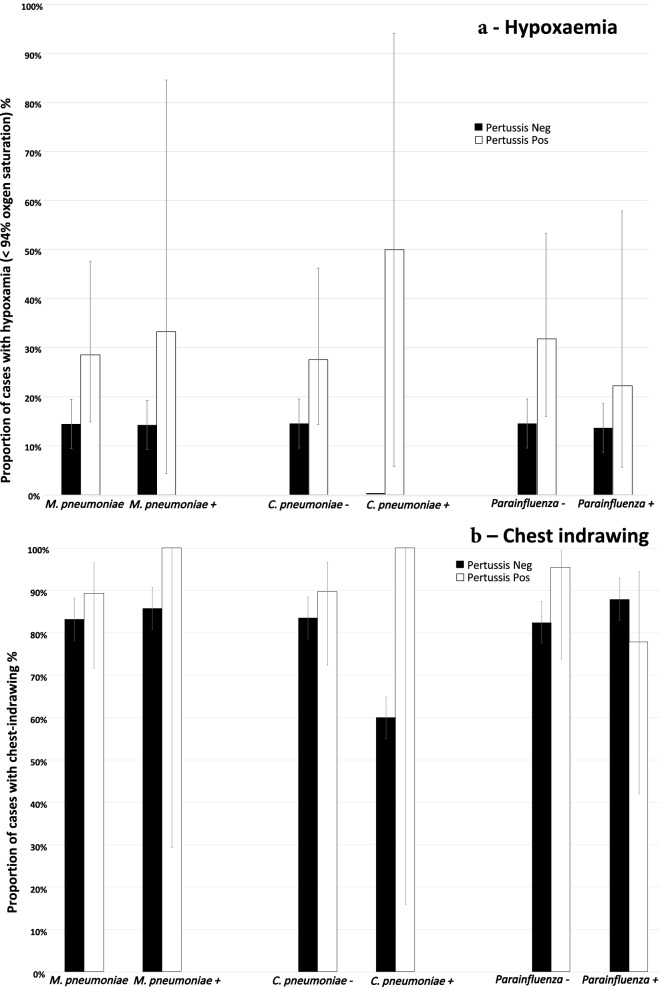
Proportion of children with hypoxaemia and chest indrawing by presence of co-detected organisms that were strongly associated with pertussis.

The same pattern was noted with the two bacterial organisms whose detection was strongly associated with *B. pertussis* (Fig. 2). A higher risk of severe disease was seen when *B. pertussis* was detected with each organism than when *B. pertussis* or each of the bacteria was detected on its own. No hypoxaemia was noted in any of the *C. pneumoniae* positive children who did not have *B. pertussis*. With parainfluenza viruses, this pattern was noted only with respect to hypoxaemia but not with chest indrawing. The small sample sizes of each stratum were not sufficient to allow for meaningful formal assessment of strength of association.

The median WCC was 13.8 (IQR 9.8–16.3) × 10^3^ cells/mL in children without confirmed B pertussis compared to 13.8 (10.4–17.0) × 10^3^ cells/mL in those with confirmed infection; P = 0.421, while children without confirmed B. pertussis had median C-reactive protein levels of 11.3 (IQR 2.7–31.5) mg/L compared to 8.4 (IQR 2–37) mg/L in those with confirmed infection; P = 0.5704.

**Figure 3:**
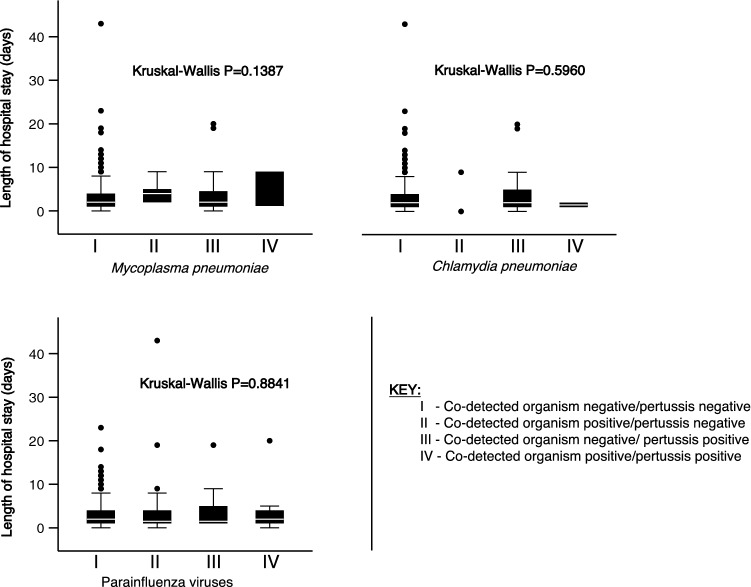
Length of hospital stay by presence of co-detected organisms strongly associated with pertussis.

The median length of hospital stay was similar in children with confirmed *B. pertussis* [2 (IQR 1–5) days] and children testing negative [2 (IQR 1–4) days]; P = 0.522. The co-detection of organisms independently associated with pertussis did not have an effect on the length of hospital stay (Fig. 3).

## Discussion

This study demonstrates a high prevalence of bacteria and viruses involved in LRTI in children requiring hospitalization for severe disease in a low- and middle-income country (LMIC) setting. The study also shows that only a few of these organisms are specifically associated with *B. pertussis* co-detection*.* The detection of *B. pertussis* was however strongly associated with a higher number of co-detected potential respiratory pathogens, specifically bacterial organisms. Although most of the studied potential respiratory pathogens did not show association with the presence of *B. pertussis*, three, namely *C. pneumoniae, M. pneumoniae* and parainfluenza viruses, were independently associated with being co-detected with *B. pertussis*. In addition, the study shows some evidence, albeit weak, that the co-detection of *B. pertussis*, together with these three organisms may be a risk for severe illness.

The finding of other pathogens in a respiratory tract specimen together with *B. pertussis* is not by itself remarkable. As noted earlier, several studies have shown that multiple potential pathogens are frequently identified from children with respiratory infections, including pertussis^4,23^. What is of interest, however, is the finding of higher numbers of potential pathogens, in particular bacterial, significantly associated with the presence of confirmed *B. pertussis*; and the association of specific organisms with confirmed *B. pertussis*.

Participants with *B. pertussis* had more organisms detected in their sputum samples, compared to those without. This association was significant for bacterial pathogens, a finding which had an impact also on association with overall total number of all detected organisms. In addition, patients with *B. pertussis* had a fourfold increase in the risk for detection of either *C. pneumoniae* or *M. pneumoniae*, as well as twice the risk to detect parainfluenza viruses.

The finding of *M. pneumoniae* in the upper respiratory tract of children in the Netherlands was found not to be associated with clinical disease^24^. The study reported prevalence of 21% and 16% in asymptomatic and symptomatic children, respectively. This shows that carriage of *M. pneumoniae* may be quite common. Unlike in the current study, the mentioned study used an upper respiratory tract sample, and did not investigate for *B. pertussis*. Similar to our study, a Swiss study found no association between *B. pertussis* and detection of viruses via PCR^25^. Again, unlike in our study, a nasopharyngeal specimen was used and not an IS sample.

In this study, although the number of viruses associated with *B. pertussis* was marginally higher, this finding was not of statistical significance. This is in keeping with findings of other studies that show little association between *B. pertussis* and viral infections^25^. Specifically, our study showed a negative correlation between *B. pertussis* and *RSV* although this finding was also not of statistical significance. This pattern was however in keeping with published literature where a negative association was noted between *RSV* and pertussis or between pertussis and bronchiolitis, a disease mainly attributable to *RSV*^26,27^. We did however, find independent association between detection of parainfluenza viruses and the presence of *B. pertussis*.

Children with pertussis generally showed more severe clinical illness (hypoxaemia, chest-indrawing and need for high dependency care). Although these findings were not all statistically significant due to the small numbers, the pattern was consistent. Children who had both strongly associated organisms and *B. pertussis* detected showed additional risk of severe disease, again without statistical significance. It is of note that the three organisms independently associated with *B. pertussis* are associated with ‘atypical’ or interstitial pneumonia which commonly presents with impaired oxygenation. There was no difference in mortality (study registered no deaths) and length of hospital stay.

There is evidence associating *B. pertussis* with pneumonia in children^4^. Although the first vaccine specifically targeting pneumonia was only introduced with the registration of a vaccine against *Haemophilus influenzae b* in 1985, the decline in pneumonia-associated mortality in the United States was noted three decades earlier^28^. This decline can not be fully explained by the improvement in quality of health care alone, and may in part be explained by the rapid decline in reported pertussis cases following the introduction of DPT in the 1940s. The decline in pneumonia-associated mortality mirrors that of pertussis over the period.

Due to the small sample size, our study was limited both in exploring the effect of other factors such as HIV infection and in its ability to establish strong evidence for some associations, even where patterns suggested a correlation. In some instances, in which strong association was demonstrated, the precision of the estimated risk was low due to the same limited sample size. In addition, the study is limited only to the organisms included in the multiplex PCR, noting also that distinguishing between benign colonisation and pathological infection from PCR detection of an organism in the respiratory tract remains a challenge^3^. As such these findings must be interpreted with caution.

Further well-powered studies or creative metanalytical systematic reviews are required to study this phenomenon further.

Partial and waning immunity, especially in individuals vaccinated with aP vaccines, has been shown to lead to pertussis not presenting in a classical manner^29–31^. This atypical nature of presentation may lead to the diagnosis being missed unless a high index of suspicion is maintained. South Africa, where this study was conducted, changed from wP to aP containing vaccines in early 2009^19^.

Less than 10% of children with confirmed *B. pertussis* in this study were clinically suspected of having possible pertussis, which highlights the need for laboratory support in the diagnosis of the disease. Where the diagnosis needs confirmation, the use of an additional IS specimen seems to improve detection rates^4,15^.

As we have shown, in this cohort of children receiving at least one dose of the pertussis vaccine was strongly associated with reduced risk of *B. pertussis*. Improving vaccine coverage for pertussis remains the most affordable and effective tool to decrease the incidence of pertussis; and may indirectly also reduce that of severe LRTI due to associated pathogens, as suggested by the data presented in this study. This is particularly important in LMICs where childhood pneumonia still accounts for up to 15% of under-five mortality^32^.

## Data Availability

All data analysed during the current study have been presented in this manuscript, but the corresponding datasets generated are available from the corresponding authors on reasonable request.
